# qSanger: Quantification of Genetic Variants in Bacterial Cultures by Sanger Sequencing

**DOI:** 10.34133/bdr.0007

**Published:** 2023-02-07

**Authors:** Satya Prakash, Adrian Racovita, Teresa Petrucci, Roberto Galizi, Alfonso Jaramillo

**Affiliations:** ^1^ School of Life Sciences, University of Warwick, Coventry, UK.; ^2^ De Novo Synthetic Biology Lab, I2SysBio, CSIC-University of Valencia, Paterna, Spain.; ^3^ Department of Biotechnology, Chemistry and Pharmacy, University of Siena, Siena, Italy.; ^4^ Centre for Applied Entomology and Parasitology, School of Life Sciences, Keele University, Keele, UK.

## Abstract

Genetic variations such as mutations and recombinations arise spontaneously in all cultured organisms. Although it is possible to identify nonneutral mutations by selection or counterselection, the identification of neutral mutations in a heterogeneous population usually requires expensive and time-consuming methods such as quantitative or droplet polymerase chain reaction and high-throughput sequencing. Neutral mutations could even become dominant under changing environmental conditions enforcing transitory selection or counterselection. We propose a novel method, which we called qSanger, to quantify DNA using amplitude ratios of aligned electropherogram peaks from mixed Sanger sequencing reads. Plasmids expressing enhanced green fluorescent protein and mCherry fluorescent markers were used to validate qSanger both in vitro and in cotransformed *Escherichia coli* via quantitative polymerase chain reaction and fluorescence quantifications. We show that qSanger allows the quantification of genetic variants, including single-base natural polymorphisms or de novo mutations, from mixed Sanger sequencing reads, with substantial reduction of labor and costs compared to canonical approaches.

## Introduction

Genetic variations such as mutations, recombinations, transpositions, or other lateral transfer events occurring in growing organisms are often considered nonneutral and therefore reaching fixation in the population. Neutral mutations will only contribute to a negligible proportion of the total population. However, if the mutations are not neutral, their proportion could noticeably increase or decrease in the population, depending on the advantages or disadvantages provided by the genetic variant. Transient neutrality has been successfully applied to create genetic memory systems [1,2]. These mutations could be quantified genotypically, e.g., by quantitative polymerase chain reaction (qPCR) and sequencing methods, or phenotypically, e.g., using genetic markers. One of the fastest and cheapest sequencing methods to determine unknown nucleotide sequences of DNA is Sanger sequencing. However, Sanger sequencing from DNA extracted from a heterogeneous population produces an electropherogram with multiple traces that are generally difficult to utilize without further processing.

The quantification of ratios of mutated and wild-type DNA sequences has seen applications in the fields of diagnosis [3] as well as drug resistance in bacterial populations [4]. Relative quantification of genetic mutations can be used for the early diagnosis of disease and the development of efficient personalized treatment procedures. For example, ratios between methylated and unmethylated genes are associated with the onset of cancer [3] as well as the quantification of mitochondrial heteroplasmy [5]. Genetic variant quantification has also been successfully used to determine the proportion of bacteria strains resistant to antimicrobial drugs in a multistrain community [4]. Recently, Sanger sequencing was used to identify and measure the frequency of indel mutations following CRISPR-Cas9 gene editing [6,7].

Sanger sequencing is a cost-effective and reliable method frequently used for plasmid DNA sequencing. The DNA undergoes a linear amplification process that creates DNA segments of varying sizes [8], where each new DNA fragment produced is labeled with a fluorescent color specific to the 3′ end base. Subsequently, the DNA segments are sorted based on length through capillary electrophoresis. The data are output as an electropherogram, where the fluorescent signal associated to each base is shown as a function of a time variable correlating to the movement of each DNA fragment through the gel. This produces a time-dependent 4-dimensional vector called a trace, which is used to infer the composition of a mixture using optimization methods. Unfortunately, the accuracy is not enough to quantify plasmid mixtures. A possible alternative would be the use of qPCR, which can provide precise measurements of the individual copy numbers of the mutated and unmutated genes [9]. However, qPCR has the following disadvantages: high cost and complex optimization to discriminate independent genetic variants.

Previous work relied on the use of universal marker genes in order to compute the DNA ratio [10]. The procedure involves the use of PCR to amplify the marker genes from a multistrain bacterial population, followed by the Sanger sequencing of the DNA mixture to generate electropherograms containing a linear combination of traces from each individual strain. This is then used to compute the fraction of each marker gene sequence from the total DNA. Another work kept the ambiguity at positions with overlapping nucleotides by reading the nucleotide sequence [11]. This allowed for the identification of all constituent sequences in the DNA mixture, which leads to an accurate characterization of the cocultures of 2 and 3 strains by comparing the identified sequences to known databases of universal marker genes.

In this work, we propose qSanger, a new methodology that uses the data from Sanger sequencing to quantify the constituent ratio of genetic variations in a bacterial culture. We validated qSanger with plasmid and coculture ratios using both qPCR and fluorescence-based assays.

## Materials and Methods

### Strains and growth media

All strains used in this study were grown aerobically at 37 °C in 14-ml culture tubes in Luria-Bertani (LB) liquid media or on LB 1.5% w/v agar. All cloning experiments were done using the *Escherichia coli* Top10 (genotype [F-mcrAΔ(mrr-hsdRMS-mcrBC) *Φ*80 lacZΔM15ΔlacX74 recA1 endA1 araΔ139 Δ (ara, leu)7697 galU galK *λ*-rpsL (StrR) nupG]). The bacteria were grown in LB media at 37 °C and 200 revolutions per minute (rpm) in the presence of antibiotics (final concentrations of 80 μg/ml for carbenicillin, 50 μg/ml for kanamycin, 100 μg/ml for ampicillin, and 34 μg/ml for chloramphenicol). The chloramphenicol resistance from the genome of Marionette DH10B was cured by electroporating the pE-FLP plasmid [12] into competent cells followed by selection on LB agar with 50 μg/ml ampicillin at 30 °C for 24 h.

### Plasmid mixture cotransformation

All plasmid constructs used in this study are listed in Table S1. The plasmids were obtained from a previous work [2]. Competent cells of *E. coli* Marionette DH10B were transformed with 80 ng of P1 and 80 ng of P2 plasmids. Antibiotic selection was carried out on LB agar with 60 μg/ml ampicillin, 17.5 μg/ml kanamycin, and 11 μg/ml chloramphenicol as well as the cognate chemical inducer for the plasmid promoter that controls the antibiotic resistance genes necessary for selection. The concentrations of the inducer for the transformations are included in Table S2. Single colonies were picked and inoculated into 2 ml of LB media with 100 μg/ml ampicillin and grown overnight at 37 °C and 200 rpm. Glycerol stocks (50% v/v) were made and stored at −80 °C.

### Plasmid mixture characterization by Sanger sequencing

Bacteria glycerol stocks were inoculated into 5 ml of LB media with 100 μg/ml ampicillin. The cells were grown overnight at 37 °C and 200 rpm. The plasmid DNA was extracted with the GeneJET Plasmid Miniprep Kit (Thermo Scientific, UK). Minipreps from P1 and P2 cells and from cotransformed strains were subjected to Sanger sequencing (GATC, Germany) using the primers included in Table 2. The CamR primer was designed to anneal to the chloramphenicol resistance gene in a region common to both the P1 and P2 plasmids, regardless of the promoter carried. The resulting P1:P2 electropherogram traces of the Sanger sequencing were used for the computational analysis. For the analysis of 2-strain colonies of bacteria cotransformed with P1 and P2 plasmids, the DNA mixture was sequenced using the pBAD-F and pCin-F forward primers (Table 2), which were designed to specifically anneal to the promoter (either the pBAD or pCin promoters). For 2-strain colonies, it was necessary to sequence the mCherry and enhanced green fluorescent protein (EGFP) genes with forward primer designed to align specifically to only one fluorescent reporter gene (either the mCherry-F or enhanced green fluorescent protein (EGFP)-F primers in Table 2) in order to align the P1:P2 electropherograms.

### Plasmid mixture fluorescence characterization in liquid medium

Bacteria glycerol stocks were inoculated into 2 ml of LB media with 100 μg/ml ampicillin. The cells were grown overnight at 37 °C and 200 rpm. The overnight culture was refreshed by a 2,000-fold dilution in M9 medium (1× M9 salts, 100 μM CaCl_2_, 2 mM MgSO_4_, 10 μM FeSO_4_, glycerol 0.8% v/v, supplemented with casamino acids 0.2% w/v, 1 μg/ml thiamine, 20 g/ml uracil, and 30 μg/ml leucine, using NaOH to adjust the pH to 7.4) with cognate inducers and 80 μg/ml carbenicillin followed by 3 h of growth at 37 °C and 200 rpm. The concentrations of the inducer for the transformations are included in Table S2. After growth, 200 μl of culture samples was added to 96-well plates (Corning Costar). The cells were characterized using an Infinite F500 microplate reader (Tecan) at 37 °C and 200 rpm. The optical density at 600-nm absorbance (OD_600_) was assayed with an automated protocol of repeated absorbance measurements with a 600-nm absorbance filter, and measurements were taken every 17 min for 18 h. Red and green fluorescence values were also measured every 17 min for 18 h (580/20 nm excitation and 635/35 nm emission for mCherry, 465/35 nm excitation and 530/25 nm emission for EGFP). All measurements were conducted using 3 technical and 3 biological replicates. The plasmid ratios (*ω*) of bacteria cotransformed with the P1 and P2 plasmids characterized in liquid medium were calculated using Eq. 1, where *ω* is calculated from the red and green fluorescence values of fully induced cotransformed bacteria. The variables of Eq. 1 are defined as follows: R = red fluorescence, G = green fluorescence. R^P1^ is the red fluorescence value of fully induced P1 cells, and G^P2^ is the green fluorescence value of fully induced P2 cells. The equation was derived and validated in a recent work [2].

$$\omega =\frac{1}{1+\lambda (\frac{G}{R}-\beta )},\text{where}\;\lambda =\frac{R^{P1}}{G^{P2}}\;\text{and}\;\beta =\frac{G^{P1}}{R^{P1}}$$ (1)

The *λ* and *β* values were computed for each promoter from the red and green fluorescence values of the P1 and P2 cells characterized in liquid medium (Table 1).

**Table 1.** Parameters for P1:P2 ratio calculation from fluorescence data.

Promoter	*λ*	*β*
pSal	1.05	0.07
pTet	0.52	0.14
pBet	0.46	0.05
pBAD	0.98	0.06
pLux	0.84	0.19
pVan	0.64	0.01
pTtg	0.11	0.13
pTac	0.56	0.02

### Plasmid mixture characterization by qPCR

Glycerol stocks of bacteria cotransformed with the P1 and P2 plasmids were inoculated from −80 °C in 5 ml of LB media with ampicillin (100 μg/ml final concentration). The cells were grown overnight (18 h) at 37 °C in a shaker incubator set at 200 rpm. The plasmid DNA was extracted using the GeneJET Plasmid Miniprep Kit (Thermo Scientific UK). The samples were analyzed with quantitative real-time PCR (qRT-PCR) analyses conducted on the StepOnePlus™ Real-Time PCR System (Thermo Scientific, UK) with the Fast SYBR-Green master mix (Thermo Scientific, UK). The P1 plasmids of all promoters were quantified using the RG1083 and RG1084 primers (Table 2), which were designed to specifically anneal to a region in the mCherry gene (the only sequence unique to the P1 plasmids). The P2 plasmids were quantified using the RG1087 and RG1088 primers (Table 2), which were designed to specifically anneal to a region in the EGFP gene only found in the P2 plasmid. The RG1080 and RG1082 primers anneal to a backbone region common to both the P1 and P2 plasmids and served as a control for the quantification of the P1:P2 intracellular ratio.

**Table 2.** Primers used in this study.

Primer	Sequence (5′ to 3′)
CamR	TAACTGACTGAAATGCCTCAAAATG
pCin-F	CCCTTTGTGCGTCCAAACGGAC
pBAD-F	CACATTGATTATTTGCACGGCGTC
EGFP-F	TGTTCACCGGGGTGGTGCCCATC
mCherry-F	GCGAGGAGGATAACATGGCCA
RG1083	CCTGTCCCCTCAGTTCATGT
RG1084	GTCCTCGAAGTTCATCACGC
RG1087	GACGACGGCAACTACAAGAC
RG1088	TCCTTGAAGTCGATGCCCTT
RG1080	CTGACCGCGTTTCTGCATAA
RG1082	GGCATGGTGGTATCACGTTC

### Statistical analysis

All measurements were taken from distinct samples and the experiments were not randomized. All experiments were conducted using 3 technical and 3 biological replicates. All Pearson’s *R* coefficients were calculated through linear regression analysis. Nonparametric analysis of similarities (ANOSIM) tests (Vegan R package) [13] were used to infer the significance of all data and the similarity of 2 sets of data produced through different measurements.

## Results and Discussion

### Algorithm for the quantification of electropherograms

qSanger aims to accurately compute the ratio of genetic variants in a bacterial population. As a proof of concept, we tested qSanger using bacterial colonies cotransformed with 2 plasmids including distinct fluorescent markers: mCherry in P1 and EGFP in P2 (Fig. 7). Ungapped global alignment of a 700-bp sequence within the EGFP and mCherry resulted in 37.6% identity. Nonetheless, qSanger can also be used for relative quantification of sequences even if sharing 100% identity, if it is possible to place the same primer at different shifted locations, resulting in an alignment of shifted sequences. In general, this alignment would only produce a 25% identity of matches due to the likelihood of random nucleotide alignments. Based on this genetic variation between P1 and P2, the Sanger sequencing of cotransformed cells generates an electropherogram with multiple traces that we call P1:P2 trace. To calculate the relative plasmid ratio, the mixed electropherogram traces were aligned against traces obtained from the sequencing of P1 and P2 using the standard global alignment function from Biopython [14]. The DNA traces produced by Sanger sequencing are not aligned because the DNA fragments travel at different speeds through the capillary gel during the reaction. Thus, we stretched the time of P1 and P2 traces to align with the P1:P2 trace at the anchoring points given by positions where nucleotides are the same in both P1 and P2 traces (and therefore the P1:P2 electropherogram shows a single peak). Although the quality of the electropherograms can vary from sample to sample, our method is based on the comparison between 2 reads obtained from a unique sequencing reaction thereby averaging potential variations derived from lower-quality scores.

qSanger first aligns the electropherogram traces via base-calling and a global sequence alignment against the P1 and P2 reads. The first 20 nucleotides are discarded as they correspond to a region where the mCherry and EGFP sequences are identical. In addition, the nucleotide calls after the first 1,000 are discarded as the quality of the electropherograms decreases past this point. The identical nucleotides of the P1 and P2 sequences at a given time point were used as anchor points to normalize the traces to account for different signal decays of the amplitudes. The anchor points are used to stretch the time and amplitude of the P1 and P2 sequences such that they align with the P1:P2 trace at a minimum of 5 anchoring points. The P1:P2 plasmid ratio in cotransformed cells is computed by evaluating the ratio of peaks between 2 consecutive anchoring points at nucleotide positions where the aligned EGFP and mCherry sequences are different and therefore the P1:P2 trace shows 2 peaks. qSanger only considers peaks between positions 20 and 600 to ensure the highest reads quality is used. Mismatches are defined as regions where neither peak in the P1:P2 trace corresponds to the same nucleotide from the P1 and P2 reads.

**Fig. 1.**
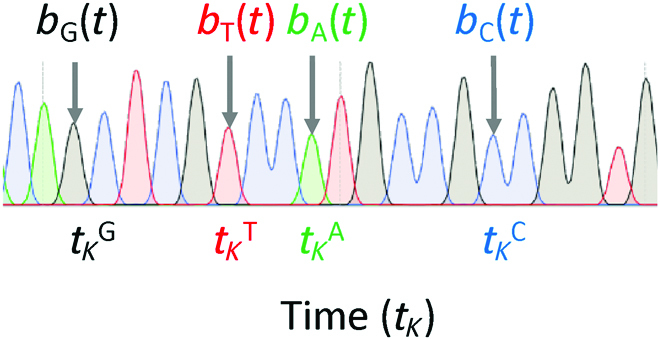
Representation of Sanger sequencing traces corresponding to the nucleotides with the highest amplitude (black = guanine, green = adenine, red = thymine, and blue = cytosine) at their respective time point (*t*).

Nucleotide sequences from mCherry and EGFP traces are generated by recording each chromatogram and its specific time point (*t*). Sanger sequencing chromatograms consist of a 4-dimensional vector containing the signal intensities at equally spaced time intervals during the sequencing run, which are visualized as peaks (Fig. 1). We retrieve the sequence and signal intensities of the peaks using the Biopython Bio.SeqIO package [14].

We define *b_i_* (*t*) as the trace of nucleotide *i* (Fig. 1 and Eq. 2) and *t_K_* as the time at each trace peak *K*.

$$\left\{ \begin{array}{c} b_{i}^{M}(t)=\text{temporal trace of the}\;P1:P2\;\text{trace nucleotide}\;i\;(i=G,A,T,C),\\ b_{i}^{P1}(t)=\text{temporal trace of the}\;P1\;\text{trace nucleotide}\;i\;(i=G,A,T,C),\\ b_{i}^{P2}(t)=\text{temporal trace of the}\;P2\;\text{trace nucleotide}\;i\;(i=G,A,T,C),\\ b^{M1}(t)=\text{temporal trace of the}\;P1:P2\;\text{trace aligned to the}\;P1\;\text{trace},\\ b^{M2}(t)=\text{temporal trace of the}\;P1:P2\;\text{trace aligned to the}\;P2\;\text{trace}. \end{array}\right.$$ (2)

We also define the nucleotide present at the peak *K* as the nucleotide with the highest trace (*S_K_*) using the following Eq. 3:

$$\left\{ \begin{array}{c} S_{K}^{P1}=\max(b_{1}^{P1}(t_{K}),\ldots ,b_{4}^{P1}(t_{K})),\\ S_{K}^{P2}=\max(b_{1}^{P2}(t_{K}),\ldots ,b_{4}^{P2}(t_{K}),\\ S_{K}^{M1}=\text{max}(b_{1}^{M1}(t_{K}),\ldots ,b_{4}^{M1}(t_{K})),\\ S_{K}^{M2}=\text{max}(b_{1}^{M2}(t_{K}),\ldots ,b_{4}^{M2}(t_{K})). \end{array}\right.$$ (3)

where max(...) returns G, A, T, or C if the highest value is the first, second, third, or fourth element. max2(...) returns G, A, T, or C if the highest value is the first, second, third, or fourth element at the second peak. We consider that there is a second peak present only if $b_{i}^{M2}(t_{K})$ ≥ 0.014 $\sum_{j}b_{j}^{M}(t_{K})$ and if $b_{i}^{M2}(t_{K})$ ≥ 50. $b_{i}^{M1}$ is the trace corresponding to the nucleotide of the P1 trace and $b_{i}^{M2}$ is the trace corresponding to the nucleotide of the P2 trace.

**Fig. 2.**
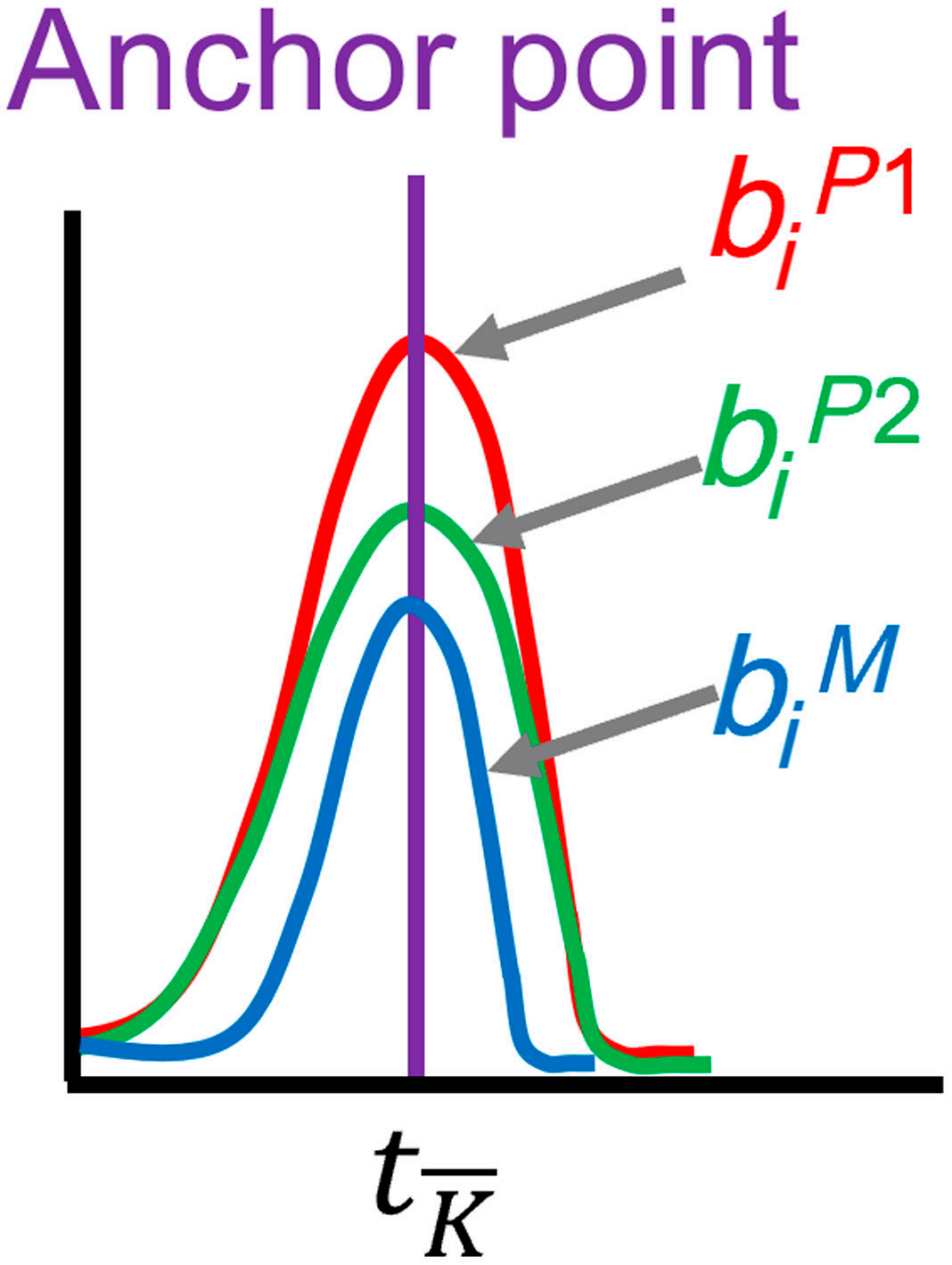
Representation of the traces *b*(*t*) around anchor points, where the same nucleotide is present in the P1 and P2 alignment $(b_{i}^{P1}$ = trace of the P1 read, $b_{i}^{P2}$ = trace of the P2 read, and $b_{i}^{M}$ = trace of the mixed read).

We use a global alignment algorithm to align the peaks of the P1:P2 traces with the P1 and P2 reads. The alignment is used to define anchor points at times $\left(t_{\bar{K}}\right)$ of identical nucleotides among the aligned P1, P2, and P1:P2 traces (Fig. 2). Anchor points are referenced using overlines $\bar{K}$ .

The alignment is assessed with a penalty function based on the matches and mismatches between the nucleotides using the following Eq. 4:

$$M_{\mathrm{mat}}=\{\begin{array}{c} 1\;\text{if}\;(S_{K}^{M1}=S_{K}^{P1}\;\text{and}\;S_{K}^{M2}=S_{K}^{P2})\;\text{or}\;(S_{K}^{M1}=S_{K}^{P1}\;\text{and}\;S_{K}^{M2}=S_{K}^{P2})\;(\text{match score for}\;1\;\text{peak}),\\ 2\;\text{if mixed read}\;\text{has}\;1\;\text{peak but}\;S_{K}^{P1}\neq S_{K}^{P2}\;\text{and}\;S_{K}^{M1}=S_{K}^{P1}\;\text{or}\;S_{K}^{M2}=S_{K}^{P2},\\ 0.75\;\text{if only one peak aligns}\;(S_{K}^{M1}=S_{K}^{P1}\;\text{and}\;S_{K}^{M2}\neq S_{K}^{P2})\;\text{or}\;(S_{K}^{M1}\neq S_{K}^{P1}\;\text{and}\;S_{K}^{M2}=S_{K}^{P2}),\\ 0.85\;\text{if}\;(S_{K}^{M1}=S_{K}^{P1}\;\text{and}\;S_{K}^{M2}=S_{K}^{P2})\;\text{or}\;(S_{K}^{M1}=S_{K}^{P1}\;\text{and}\;S_{K}^{M1}=S_{K}^{P2})\;(\text{match score for}\;2\;\text{peaks}),\\ 0\;\text{if there is a mismatch},\\ -1\;\text{if the mixed read has}\;1\;\text{or}\;2\;\text{peaks but it is aligned with a gap with P}1\;\text{and}\;P2\;\text{reads},\\ -2\;\text{if the gap is opened or extended}. \end{array}$$ (4)

**Fig. 3.**
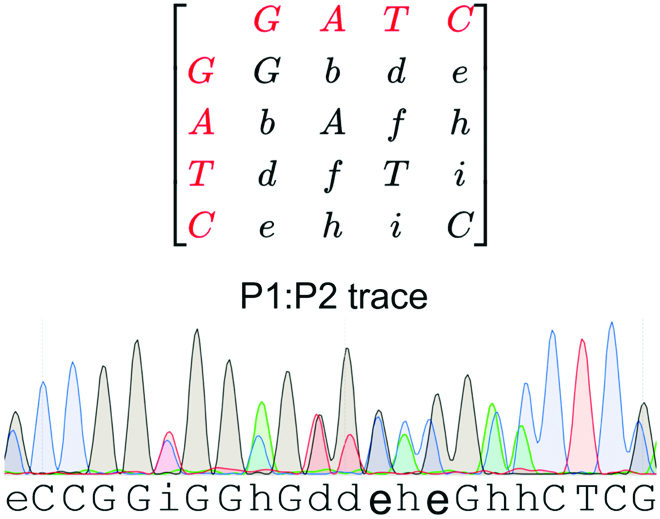
The P1:P2 trace is annotated using an extended alphabet that assigns a new letter at time points where 2 peaks are present. Top: Pairwise matrix showcasing how the extended alphabet assigns a letter to each nucleotide pair possible. Bottom: Example of electropherogram data analyzed by qSanger where new letters are placed at positions with 2 nucleotides.

In order to conduct the alignment, we choose only the anchor points $\underline{K}$ for those peaks where $S_{\underline{K}}^{P1}=S_{K_{x}}^{P2}$ and $b_{i}^{P12}(t_{\underline{K}})$ < 0.028 $\sum_{j}b_{j}^{P1}(t_{\underline{K}})$ (no second peak found in the P1 read) and $b_{i}^{P22}(t_{\underline{K}})$ < 0.028 $\sum_{j}b_{j}^{P2}(t_{\underline{K}})$ . The methodology requires at least 5 anchor points to conduct the alignment. At nucleotide positions with 2 peaks in the P1:P2 trace, the alignment is done with the highest peak. We used an extended nucleotide alphabet to generate the nucleotide sequence from P1:P2 electropherograms that assigns a different letter at positions where 2 peaks are present (Fig. 3) and the letter corresponding to the nucleotide itself in the case of a single peak.

**Fig. 4.**
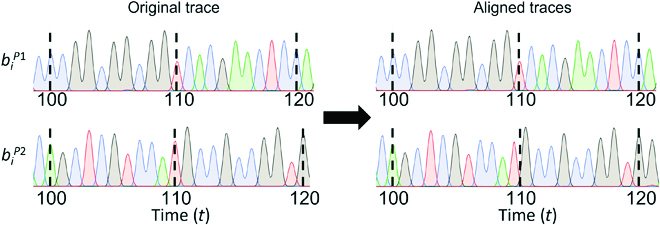
The traces are aligned using time stretching between anchor point times.

After the alignment, we create the new traces with the following time-stretching procedure: for $t_{\underline{K}}$ ≤ t < $t_{\underline{K}+1}$ , we stretch the time and amplitude of the P1 and P2 traces to align with the P1:P2 trace at the anchoring points (Fig. 4) according to the following Eq. 5:

$$\left\{ \begin{array}{c} {\bar{b}}_{i}^{P1}(t)=b_{i}^{P1}(t_{\bar{K}}^{P1}+\frac{t-t_{\bar{K}}}{t_{\bar{K}+1}-t_{\bar{K}}}(t_{\bar{K}+1}^{P1}-t_{\bar{K}}^{P1})),\\ {\bar{b}}_{i}^{P2}(t)=b_{i}^{P2}(t_{\bar{K}}^{P2}+\frac{t-t_{\bar{K}}}{t_{\bar{K}+1}-t_{\bar{K}}}(t_{\bar{K}+1}^{P2}-t_{\bar{K}}^{P2})). \end{array}\right.$$ (5)

**Fig. 5.**
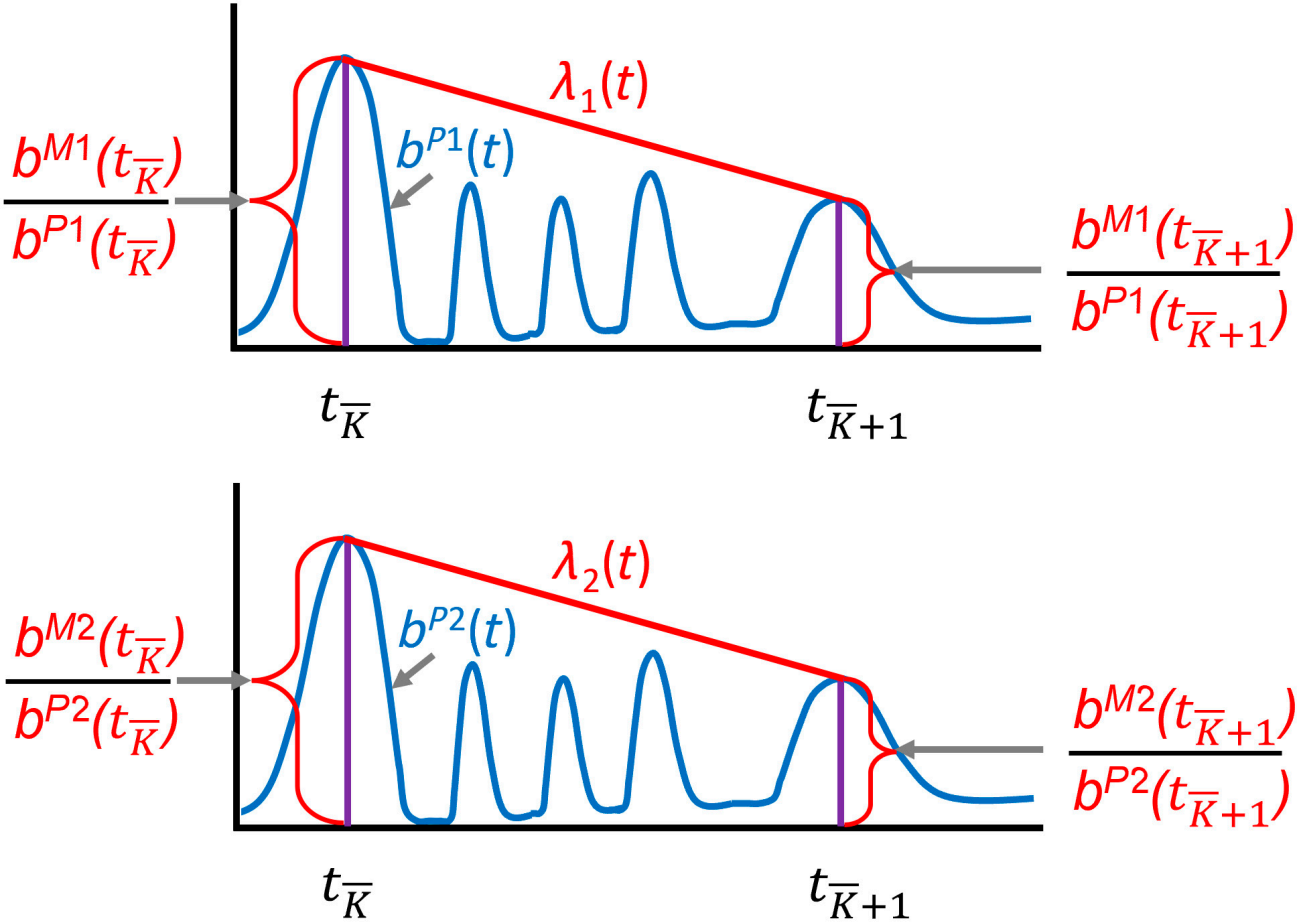
Amplitude stretching (*λ*) is computed using linear interpolation between 2 consecutive anchor points *t_K_* and *T_K+1_*.

Amplitude stretching (*λ*) is computed using the linear interpolation in Eq. 6 for $t_{\underline{K}}$ ≤ t < $t_{\underline{K}+1}$ (Fig. 5). λ_1_ corresponds to the first peak of the P1:P2 trace and λ_2_ corresponds to the second peak of the P1:P2 trace.

$$\left\{ \begin{array}{c} \lambda_{1}(t)=\frac{{\bar{b}}_{i}^{M1}(t_{\bar{K}})}{{\bar{b}}^{P1}(t_{\bar{K}})}+\frac{t-t_{\bar{K}}}{t_{\bar{K}+1}-t_{\bar{K}}}(\frac{{\bar{b}}^{M1}(t_{\bar{K}+1})}{{\bar{b}}^{P1}(t_{\bar{K}+1})}-\frac{{\bar{b}}^{M1}(t_{\bar{K}})}{{\bar{b}}^{P1}(t_{\bar{K}})}),\\ \lambda_{2}(t)=\frac{{\bar{b}}_{i}^{M2}(t_{\bar{K}})}{{\bar{b}}^{P2}(t_{\bar{K}})}+\frac{t-t_{\bar{K}}}{t_{\bar{K}+1}-t_{\bar{K}}}(\frac{{\bar{b}}^{M2}(t_{\bar{K}+1})}{{\bar{b}}^{P2}(t_{\bar{K}+1})}-\frac{{\bar{b}}^{M2}(t_{\bar{K}})}{{\bar{b}}^{P2})t_{\bar{K}})}). \end{array}\right.$$ (6)

If there is only a single peak in the P1:P2 trace, amplitude stretching is computed using Eq. 7:

$$\left\{ \begin{array}{c} \lambda_{1}(t_{\bar{K}})=\frac{b_{i}^{M1}(t_{\bar{K}})}{b_{i}^{P1}(t_{\bar{K}})},\\ \lambda_{2}(t_{\bar{K}})=\frac{b_{i}^{M2}(t_{\bar{K}})}{b_{i}^{M2}(t_{\bar{K}})}.\; \end{array}\right.$$ (7)

We infer the P1:P2 plasmid ratio (*ω*) from least squares minimization in Eq. 8, where *t_i_* (the time point of the first nucleotide, 20) and *t_f_* (the time point of the last nucleotide, 600):

$$\begin{array}{c} e=\underset{\omega \in [0,1]}{\text{min}}\int_{t_{i}}^{t_{f}}\mathrm{dt}{\left(b^{M}(t)-{\omega \lambda }_{1}(t){\bar{b}}^{P1}(t)-(1-\omega )\lambda_{2}(t){\bar{b}}^{P2}(t)\right)}^{2} \end{array}$$ (8)

**Fig. 6.**
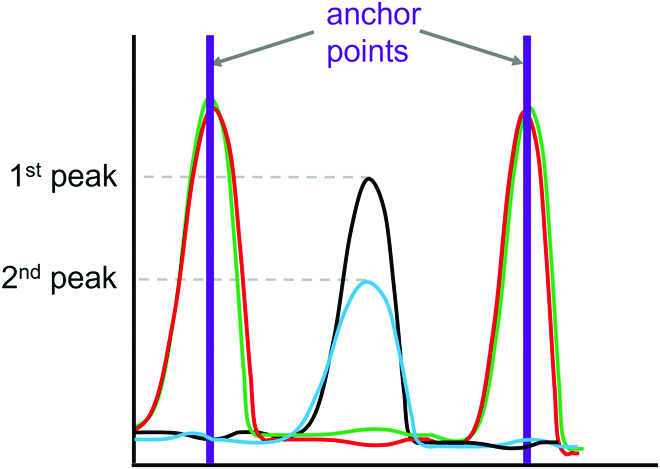
The DNA ratio is computed at time points between anchor points *t_K_* and *t*_*K*+1_, where 2 peaks are present (red = $b_{i}^{P1}$ , green = $b_{i}^{P2}$ , cyan = first peak of $b_{i}^{M}$ , and black = second peak of $b_{i}^{M}$ ).

The derivation of Eq. 9 is included in the Supplementary Text (Eq. S1). Solving Eq. S2b (Supplementary Text) for *ω* gives the P1:P2 ratio at time points where there are 2 peaks in the P1:P2 trace (Fig. 6):

$$\omega =\frac{\int_{t_{i}}^{t_{f}}\mathrm{dt}(b^{M}(t)-\lambda_{1}(t){\bar{b}}^{P1}(t))(\lambda_{1}(t){\bar{b}}^{P1}(t)-\lambda_{2}(t){\bar{b}}^{P2}(t))}{\int_{t_{i}}^{t_{f}}\mathrm{dt}{\left(\lambda_{1}(t){\bar{b}}^{P1}(t)-\lambda_{2}(t){\bar{b}}^{P2}(t)\right)}^{2}}.$$ (9)

### Experimental validation of qSanger DNA quantification

**Fig. 7.**
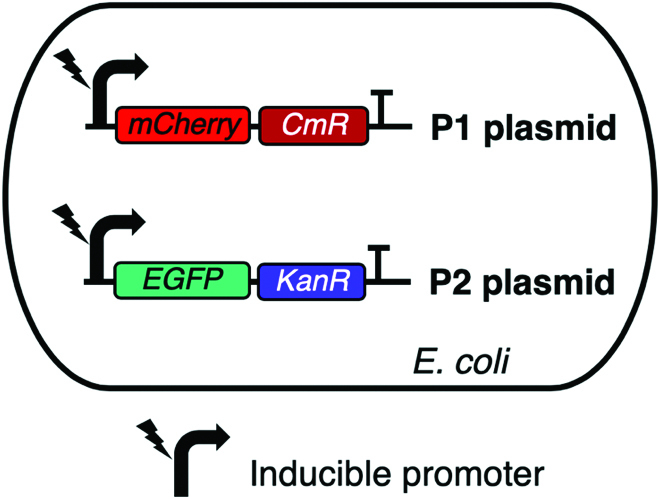
The plasmids P1 and P2 are designed to encode mCherry and EGFP fluorescence as well as the chloramphenicol (CmR) and kanamycin (KanR) antibiotic resistance genes under the same inducible promoter. These were used in a previous work to encode an inducible genetic memory system [2].

To validate qSanger method, we developed a 2-plasmid genetic system where each construct carries distinct fluorescent markers (mCherry and EGFP) allowing to use fluorescence assays to measure plasmid ratios directly in living cells. Each plasmid also carries distinct antibiotic resistance markers under the same inducible promoter to allow for artificial manipulation of plasmid ratios in a bacterial population (Fig. 7).

**Fig. 8.**
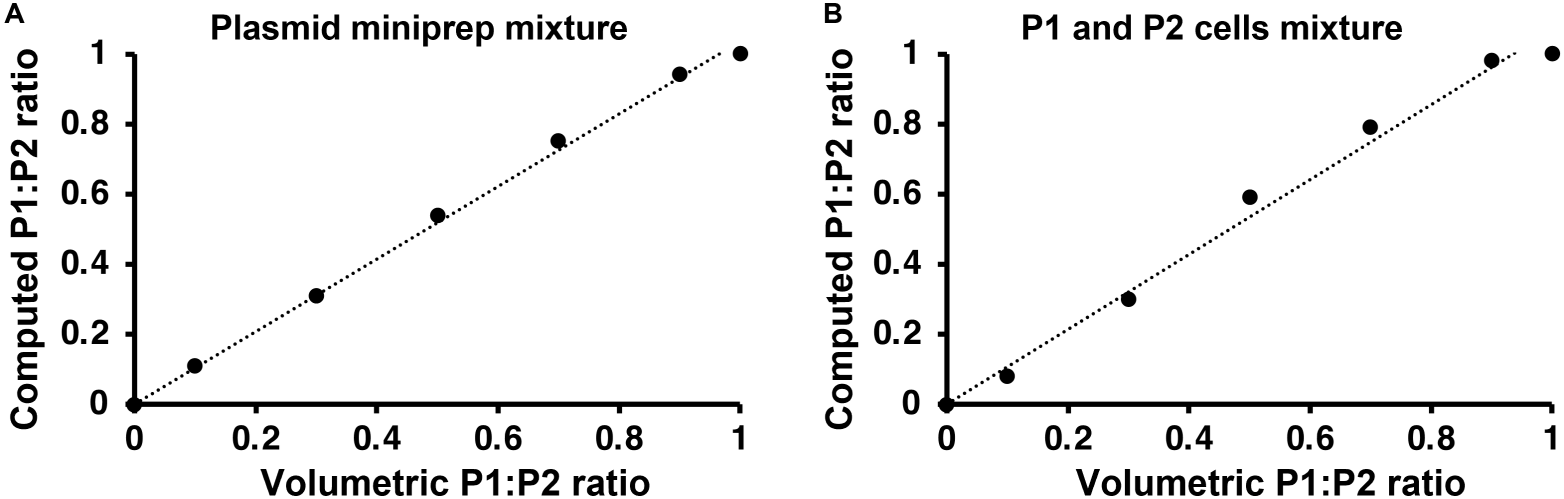
Our method can compute the plasmid DNA ratio in a predictable manner. The P1:P2 ratios computed by the software were consistent with the expected values generated by mixing P1- and P2-only cells in different molar ratios. (A) When mixing plasmid minipreps of the same DNA concentrations of P1 and P2 plasmids in different molar ratios, the ratio generated by the algorithm correlates with the expected value (*R* ^2^ = 0.99). (B) When mixing equal molar of P1- and P2-only cells, the plasmid ratios computed by the algorithm were consistent with the expected values (*R* ^2^ = 0.99).

We mixed P1 and P2 plasmids at predefined molar ratios (P1:P2 equal to 0, 0.1, 0.3, 0.5, 0.7, 0.9, and 1) and sequenced each sample using Sanger with the CamR primer (Table 2) complementary to a common sequence in the chloramphenicol resistance gene found in both P1 and P2. The resulting data were analyzed using qSanger, which was able to predict a plasmid ratio that correlated well with P1:P2 ratios generated (Fig. 8A, *R* ^2^ = 0.99).

Subsequently, we tested qSanger with DNA sequences derived from living bacteria. We transformed *E. coli* cells with either the P1 or the P2 plasmid and then mixed P1 and P2 positive cells in different molar ratios followed by plasmid purification from each mixture (Fig. 8B). Again, the P1:P2 ratio computed with our methodology were consistent with the expected plasmid ratios created by mixing the P1 and P2 cells (*R* ^2^ = 0.99).

### qSanger quantification of intracellular plasmid mixture in single-strain cultures

**Fig. 9.**
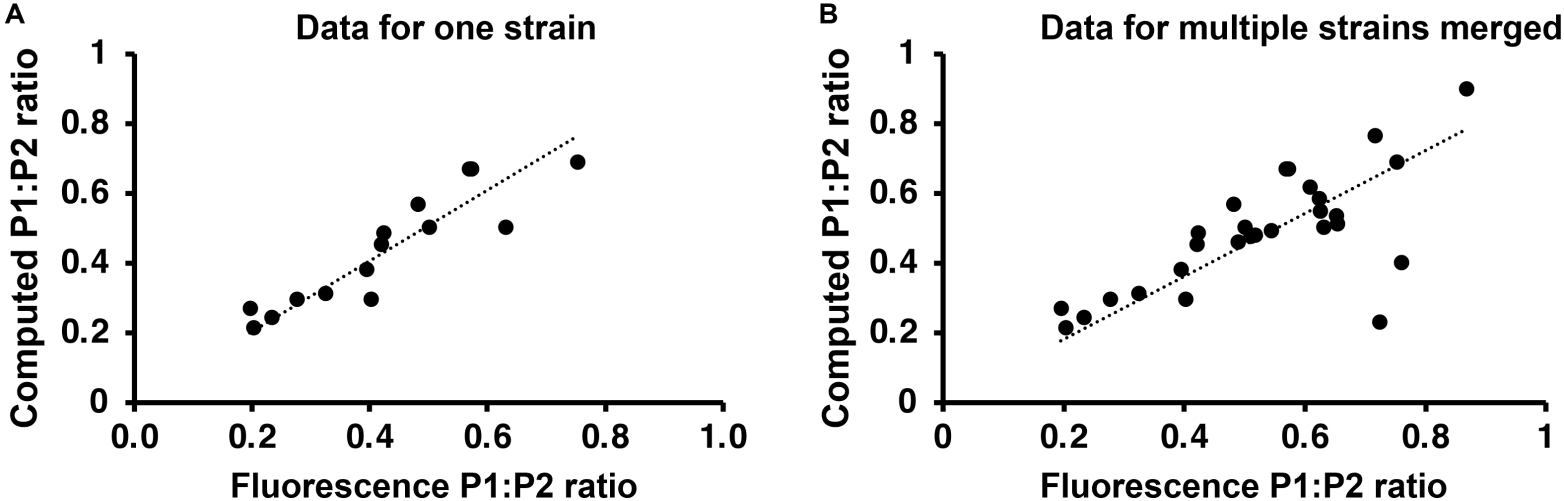
The P1:P2 ratios computed by qSanger correlate strongly with the ratios measured by fluorescence quantification. (A) The P1:P2 ratio in bacteria cotransformed with the P1 and P2 plasmids computed by qSanger was consistent with the results of fluorescence characterization in liquid medium (*R* ^2^ = 0.98). (B) Data shown for strains cotransformed with plasmids carrying different promoters (*R* ^2^ = 0.94). No statistically significant difference was found between the dataset produced by Sanger sequencing and fluorescence analysis (*P* < 0.05, ANOSIM *R* = 0.95).

**Fig. 10.**
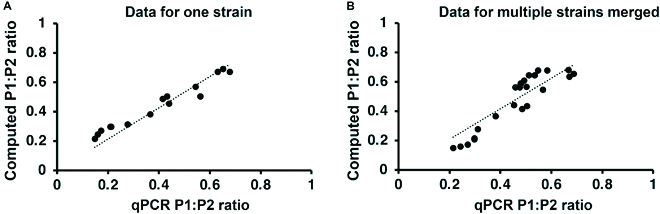
The P1:P2 ratios computed by qSanger correlate well with qPCR quantifications. (A) The P1:P2 ratio in bacteria cotransformed with the P1 and P2 plasmids computed by our methodology was consistent with the results of qPCR (*R* ^2^ = 0.98). (B) Data shown for strains cotransformed with plasmids with all promoters (*R* ^2^ = 0.94). No statistically significant difference was found between the dataset produced by Sanger sequencing and qPCR (*P* < 0.05, ANOSIM *R* = 0.96).

We also tested qSanger to measure plasmid DNA ratios from cotransformed cells by comparing qSanger results from P1:P2 electropherograms to those obtained by standard methods for DNA quantification. For this, we cotransformed bacteria with the P1 and P2 plasmids and we used Sanger sequencing compared to fluorescence quantification (Fig. 9) and qPCR assay (Fig. 10). We found that the computed P1:P2 ratios provided by qSanger were consistent with the ratios measured by a fluorescence plate reader (*R* ^2^ = 0.98 when using the data for only one strain and *R* ^2^ = 0.94 when merging the data from multiple strains) as well as those measured via qPCR (*R* ^2^ = 0.98 for P1:P2 carrying the same promoter and *R* ^2^ = 0.97 for cocultures of multiple promoters).

### DNA ratio quantification in multistrain cocultures

**Fig. 11.**
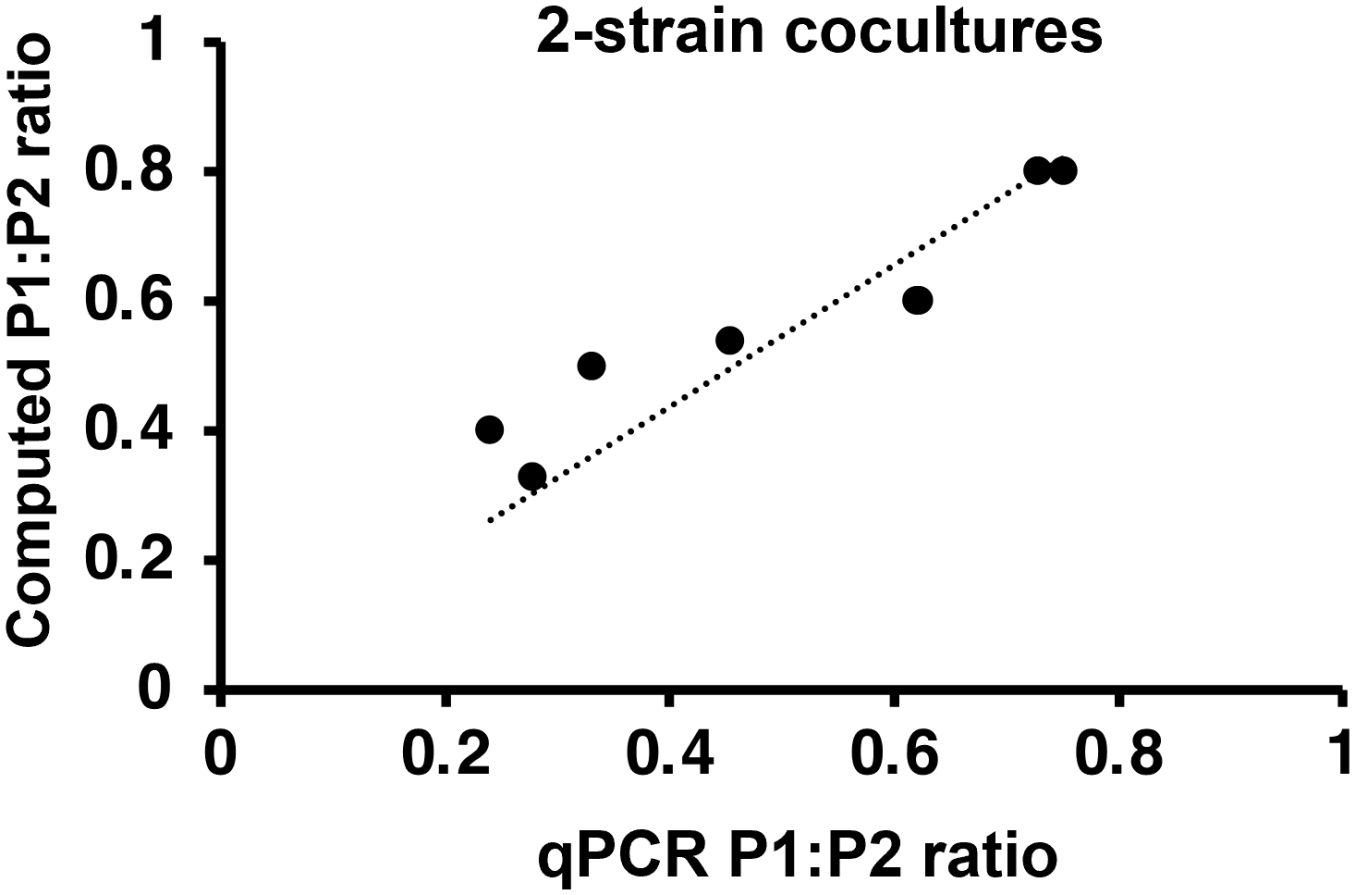
qSanger can be used to measure the intracellular plasmid DNA ratio in cocultures of 2 bacterial strains, each one cotransformed with plasmids of a different promoter (pBAD and pCin, respectively). The computed P1:P2 ratio correlates well with the ratio measured via qPCR (*R* ^2^ = 0.98). No statistically significant difference was found between the dataset produced by Sanger sequencing and qPCR (*P* < 0.05, ANOSIM *R* = 0.93).

We further tested qSanger to compute plasmid ratios from cocultures of independent bacterial strains respectively carrying both P1 and P2 under distinct promoters (pBAD and pCin). To do this, we used primers designed to anneal exclusively to one of the promoters (Table 2). This allowed to compute the P1:P2 ratio of a single strain from a coculture of 2 strains, where one strain was cotransformed with pBAD-promoter P1 and P2 plasmids and the other strain was cotransformed with pCin-promoter plasmids. The computed P1:P2 ratio was consistent with the qPCR measurements of the same cocultures (*R* ^2^ = 0.98, Fig. 11).

### DNA ratio quantification after growth in the presence of sublethal antibiotic concentrations

**Fig. 12.**
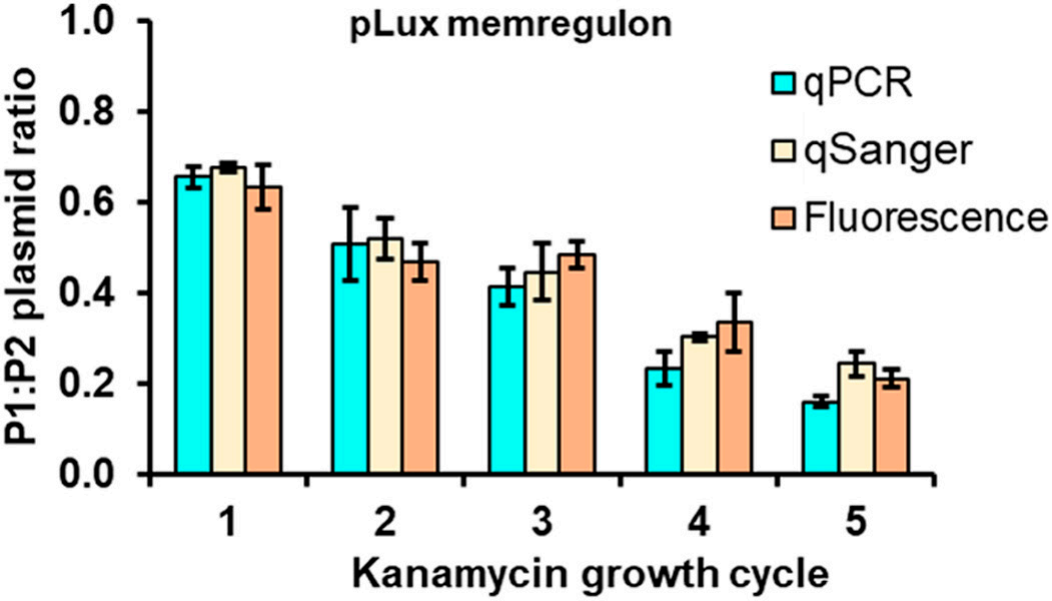
Growth cycles in the presence of kanamycin decrease the P1:P2 plasmid ratios of fully induced plasmids P1 and P2. This was confirmed by qPCR, qSanger, and fluorescence analysis. There was no statistically significant difference between the P1:P2 ratio values measured by either method (smallest *p* > 0.1).

Finally, we show that qSanger can be used to quantify plasmid ratios mimicking the evolution of a genetic variation. We simulated this by growing the strains cotransformed with P1 and P2 plasmids with the pLux promoter in sublethal kanamycin concentrations for 16 h, resulting in a gradual decrease in P1 copies and therefore a decrease in the P1:P2 ratio. The antibiotic stress was repeated for 5 consecutive iterations (in 3 biological replicates each) followed by the quantification of plasmid ratios using qPCR, qSanger, and fluorescence methods. There was no statistically significant difference in the plasmid ratios measured by either quantification method (smallest *p* > 0.1) (Fig. 12).

To further assess the applicability of this method, we tested the use of our methodology for 2 DNA sequences that differed at only one nucleotide position and complete sequence identity at all other positions. The probability to accurately infer the DNA ratio with 5% error using a single-nucleotide mutation was 30% (Fig. S1A). However, when 4 nucleotide mutations between 2 anchor points were used, the probability increased to 76% (Fig. S1B).

## Conclusion

In this study, we propose a new methodology involving the use of mixed Sanger sequencing reads to genotypically quantify the ratio of genetic variations in a bacterial culture. We validated our method by determining the plasmid DNA ratio in cocultures of single-plasmid cells (Fig. 8A, *R* ^2^ = 0.99) as well as creating synthetic ratios by mixing DNA preparations (Fig. 8B, *R* ^2^ = 0.99). In addition, the use of plasmids encoding different fluorescent markers allowed us to perform a direct comparison between measurement of genetic variants and their corresponding phenotypic outputs. Our methodology provides accurate predictions of intracellular DNA ratios as the results correlated well with those obtained by fluorescence-based quantifications (Fig. 9, *R* ^2^ = 0.94) as well as qPCR (Fig. 10, *R* ^2^ = 0.98). The methodology was also successfully applied to infer the DNA ratio in 2-strain cocultures, correlating well with qPCR measurements (Fig. 11, *R* ^2^ = 0.98).

The advantages of qSanger over technologies such as qPCR and digital droplet PCR are simplicity of use and reduced costs. Our methodology could be used to analyze mutant/nonmutant DNA ratios in cell populations after different implementations of gene editing, such as base editing [15], prime editing [16], and promoter engineering by multiplex automated genome engineering [17] or applications requiring measurement of multiple DNA or RNA sequences in the same mixture.

## Data Availability

All data are available in the main text or the supplementary materials.
