# Supplementary material for: qSanger: Quantification of Genetic Variants in Bacterial Cultures by Sanger Sequencing
Source: Biodes Res. 2023 Feb 7;5:0007. doi: 10.34133/bdr.0007 (PMC10521659; doi:10.34133/bdr.0007)
Supplement: Supplementary Materials — Supplementary Text Supplementary Figures [file bdr.0007.f1.pdf]

Supplementary Information for:

qSanger: Quantification of genetic variants in bacterial cultures by Sanger sequencing

Satya Prakash[1], Adrian Racovita[2], Teresa Petrucci[3], Roberto Galizi[4], Alfonso Jaramillo[1,2]*

[1]School of Life Sciences, University of Warwick, Coventry, UK.

[2]*De novo* Synthetic Biology Lab, I2SysBio, CSIC-University of Valencia, Paterna, Spain.

[3]Department of Biotechnology, chemistry and pharmacy, University of Siena, Siena, Italy

[4]Centre for Applied Entomology and Parasitology, School of Life Sciences, Keele University, Keele, UK.

*Corresponding author.

Supplementary Text

We integrate Equation 8 of the main text over t_i (the time point of the first nucleotide, 20) and t_i (the time point of the last nucleotide, 600):

$$\frac{de}{d\omega} = 2\int_{t_i}^{t_f} dt\ (b^M(t) - \omega\lambda_1(t)\overline{b}^{P1}(t) - (1-\omega)\lambda_2(t)\overline{b}^{P2}(t))(\lambda_1(t)\overline{b}^{P1}(t) + \lambda_2(t)\overline{b}^{P2}(t)) \quad \text{(S.1)}$$

$$\frac{de}{d\omega} = 2\int_{t_i}^{t_f} dt\ (b^M(t) - \lambda_1(t)\overline{b}^{P1}(t))(\lambda_1(t)\overline{b}^{P1}(t) + \lambda_2(t)\overline{b}^{P2}(t)) + \omega 2\int_{t_i}^{t_f} dt\ (\lambda_1(t)\overline{b}^{P1}(t) + \lambda_2(t)\overline{b}^{P2}(t))^2 \quad \text{(S.2a)}$$

$$\frac{de}{d\omega} = 2\int_{t_i}^{t_f} dt\ (b^M(t) - \lambda_1(t)\overline{b}^{P1}(t))(\lambda_1(t)\overline{b}^{P1}(t) + \lambda_2(t)\overline{b}^{P2}(t)) + \omega 2\int_{t_i}^{t_f} dt\ (\lambda_1(t)\overline{b}^{P1}(t) + \lambda_2(t)\overline{b}^{P2}(t))^2 = 0 \quad \text{(S.2b)}$$

A

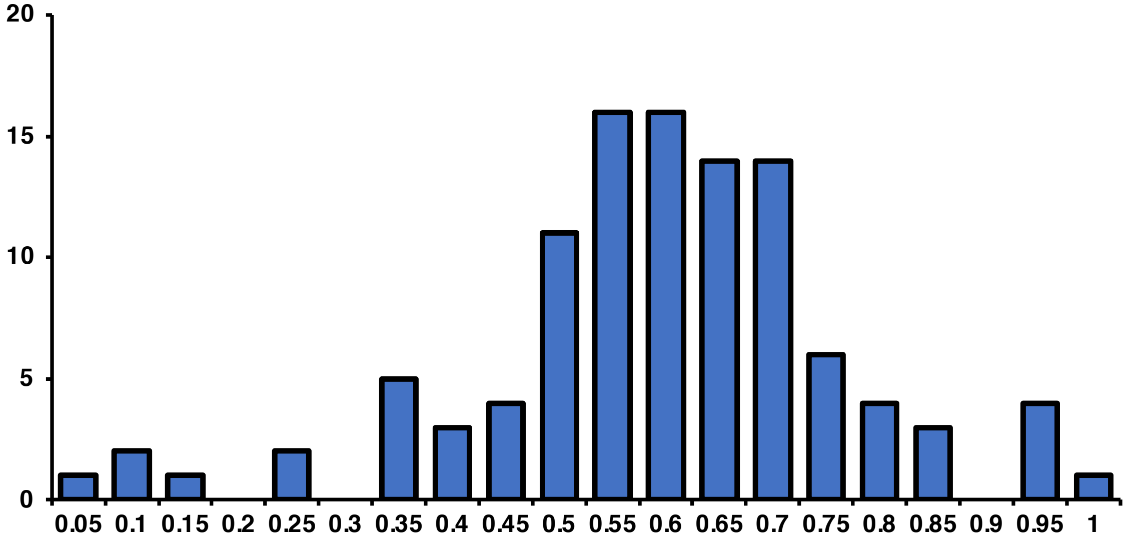

B

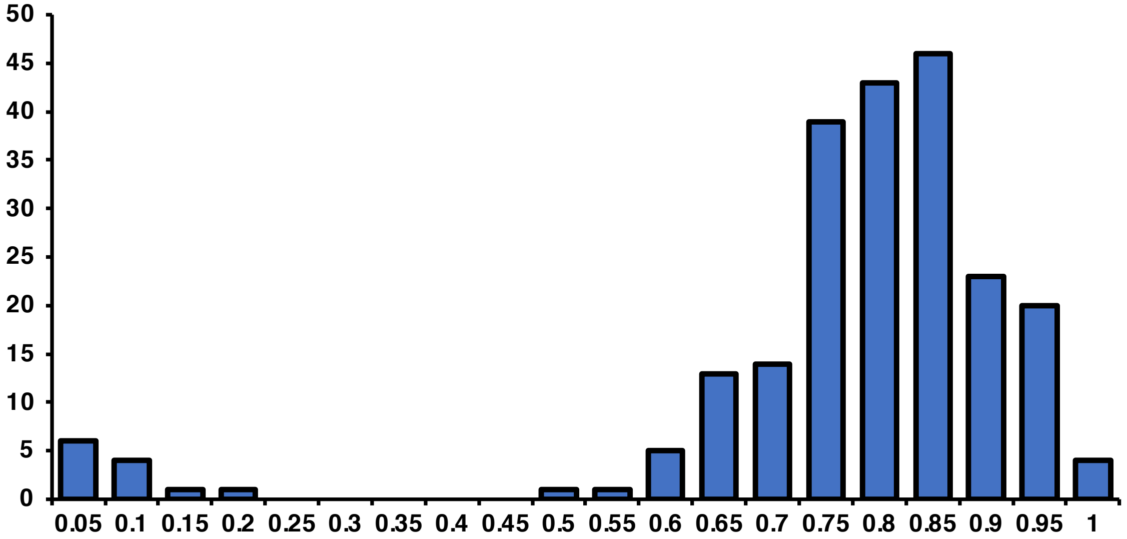

Figure S1. The DNA ratio can be accurately inferred at single-base variations. (A) The probability to accurately infer the 0.5 DNA ratio with 5% error using a single-nucleotide mutation is 30%. (B) (A) The probability to accurately infer the 0.7 DNA ratio with 5% error using the average values of 4 consecutive single-nucleotide mutations is 76%.

The following sections include the sequence alignment of the P1:P2 traces used in Fig. 6 of the main text. The alignment uses the extended alphabet from Fig. 3 of the main manuscript. Pipe characters denote anchor points.

9P1-1P2-Premixed.ab1

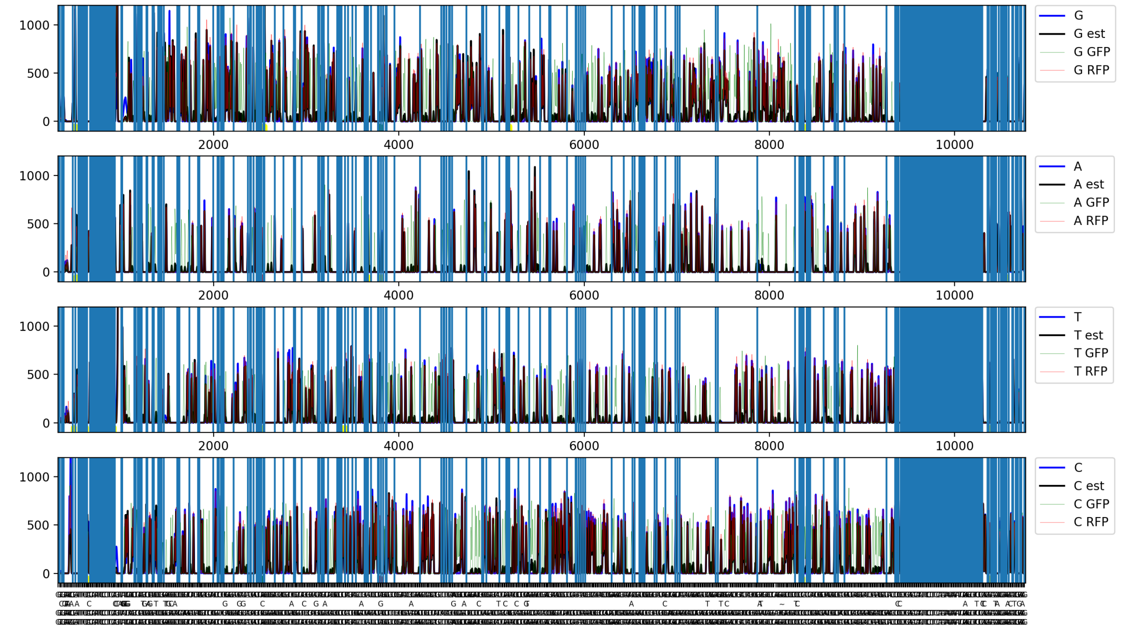


```
seq       =
fThhC||i||dffff||.||||f|||||||||h||||||||||||||||||||||hed||f|dbdee.G|GCC||
T||A||..T||..CA||C.G|||T|df|.G.CG|.C|||GGCGC|TT|GTAC||T||CACGA|GGT||A|TCC||G
|||dGGGA||T|ATG|.CAA.T|G|T||T|AC|||||.||CGCCGG|||GCT|CA||GGCTTC.|||CCT|||A.GT
GGTC|T|A.C||A|||.CG|AGTG||C||||||||TC|GC||CA|C|TC.G|||||TC|CGC||||.|G|G||CCG|
C||CGGG||ACAT|CGC.CGG||GA|GCC|CC|A|CCCAT|G|C|T||T|T||A|.ACGGGGCC|.C|GA|GG|AAG
|.GG||CC|CGC|GCTT|A.|TTG|.||||AAC|C.C|GTCCTG..A|GAGGA|TC||GGGT|A|||TCACCACGCC
|CC||CCT||AA||T|A|C|C|CGCTCCCACTTGAAGCCCT||GG|AAGGACA||T|CAA|T|b|C|GG||T||||G
CGG|G|G|T|CAC||Ae|CC|T|GA||C|T|CATGAA|TGAGG|G|CAGG|T|TCiCAGG|||A||GiA|GGe|CAC
|CTTGGT|ACCTTCAGCT||GC|GfiTGGG|GCCCTCGAGGG|G|CCCTC|C|C|ie||||||AT||||AACTCGTG
||C|TT|AC|GA|||C|CC|T||GCA|C|TG|AGCGC|TG|ACTCCTTG|TG|TGG|CA||TTATC||||i|e||||
||||||||||||||||||||||||||||||||||||||||||||||||||||||b|||||||||i||||iih||||
|||||i|b||||||||b|h|||i|||d||h|||T
seq_GFP   =
00000T......................................................................
............................................................................
...................G........................................................
............................................................................
............................................................................
............................................................................
............................................................................
............................................................................
............................................................................
............................................................................
............................................................................
.................................0
seq_RFP   =
00000T....G.................................................................
```

```
.............................................................................
.............................................................................
.............................................................................
.............................................................................
.............................................................................
.............................................................................
.............................................................................
.............................................................................
.............................................................................
.............................................................................
.................................0
```

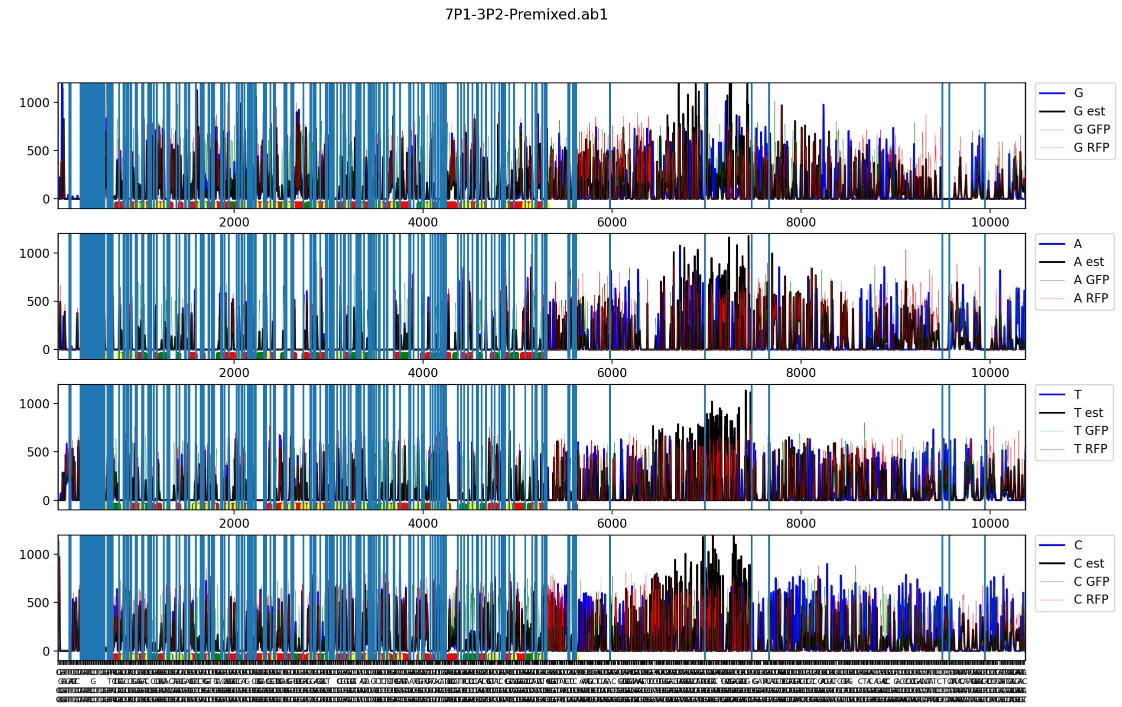


```
seq        =
dddGGG|G|dAff|||.||||f|||||||||h|||||||||||||||||||||||||||||A|||CTTGT|CA.||C
||C||TG.||A.AG||AT.||||C||.|G.CAC|AA|||CAGTA|GA|CATG||A||GCGC.|..C||.|G.G||.|
||C.CA.||C|GA.|G.GTGC|.|.||.|.G||||C||GC....|||CG.|GC||.C....A|||G.G|||TC.G.
.G.|.|...||G|||AGC|GC..||T||||||||C.|TG||.T|G|..A.|||||..|TCA||||G|T|C||..C|T
||GC.T||C...|ATGATAT||AC|TTG|GG|.|T..TA|.|.|A||C|A||T|G.GC...A.|AT|T.|.C|..C|
C.T||.A|TCG|TGCC|TT|A..|C||||...|TCA|.A.GG.GTC|..C..|.A||.C..|.|||..CG.GTCT.|
T.||.G.||.C||.|T|G|A|AA.A.GGTGCGCTCCTGGA||TA|.CTTCbG|i.|G.C|G|C|T|AAb|A||||TG
CT|C|Ti.|GTG|fC||.G|A|CG||T|AhbibCTGibCGCC|fhbGTC|Gde..bTCh|b|G||TbG|C..e.GC.
|d.G.Ae|TTG.CGGTdGd|Cb|iGA.CTdC..GeTCAG.TT||C|T.ded|.|fi|e||||biCi||bCCGGACb
.e|T|AA|TT|TG|||GiTT|e||C.C|G|CC|GCTCG|CC|GGATGGGi|CC|eCC|GG||AAChG||||||e|||
||||||h||||||||||||||||||||||||||||f|||||||h||||||f||||d|bb||i||||||eiiii|he||
|i||f||bb||||b|||||hif|i|||dbAhheii
seq_GFP   =
000000TG..G..................................................................
.............................................................................
.............................................................................
.............................................................................
.............................................................................
```

```
.............................................................................
.............................................................................
.............................................................................
.............................................................................
.............................................................................
.............................................................................
.............................000000
seq_RFP    =
000000TG..G..................................................................
.............................................................................
.............................................................................
.............................................................................
.............................................................................
.............................................................................
.............................................................................
.............................................................................
.............................................................................
.............................................................................
.............................................................................
.............................000000
```

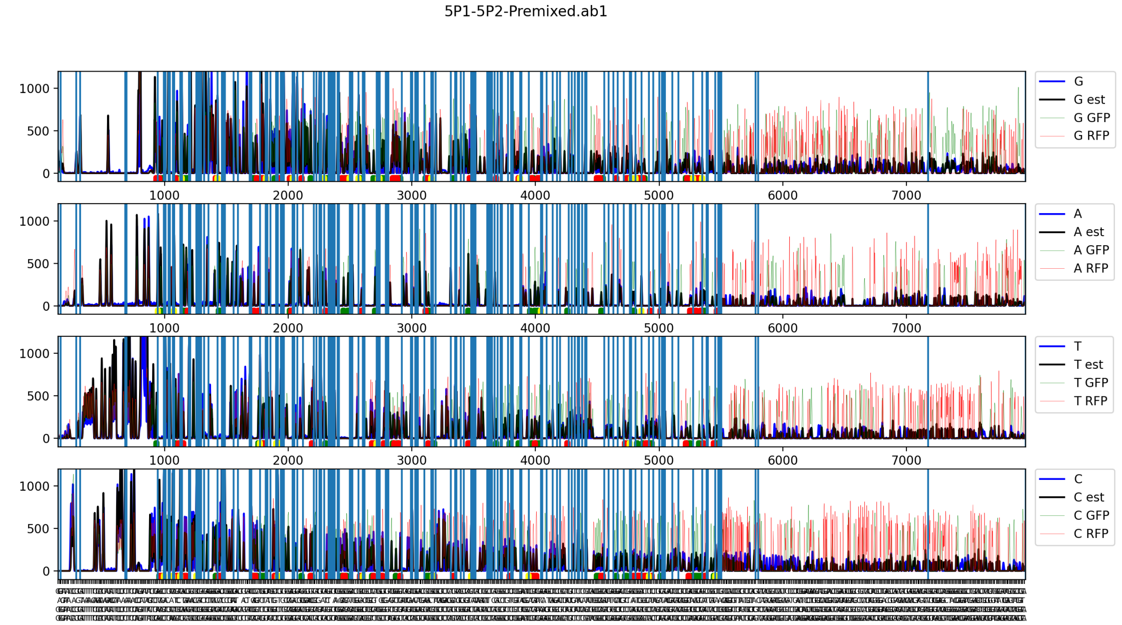


```
seq        =
ibbGeG|b|dffff||.|d|ff|fff|ihfe.hf|fbbfhiifih||f|fff|f|ihedf|f|fb.....|...|f.
||.||...||....f|...|ebd.|d.d.....|..ii|.d...b..|G|bi|.||.....|...|f.|...||.|d
|.....|b.|...d......d.f.||.|..d|||f.||......|i|...|..|e.......|||...d||......
..f.d...f|.e|b...|....||.biebdeef..|..f|..e.|....|ffdf..f...ii||.|.b.|e...|.i
|....|f....|.......|e.A|...f..|.e.....e.i.|.if.|.|i.i.........b..b..|..b...|.
..||..|...|....|..i...|.d|fb...f...i.........|.....|..i|....|.|b|ieheebieiiGi
hb|ieiidfh|iiA.ibhGebefieebCifefCiCeTheb.Ghhed.ibdeifiebe.bbiideb.b.bdieeib..
Gidiifibi.dfe.eedTb|hGeid.Ahbibh.eibhbee|fhbGdhf|deiebdehebAeGGibGeeebeebihi.
dGefehiidii.deTddbib|fidbbeT.ihidediAGeddeeeeihdedeeifieeiiiiebiiiiiebbideGfb
eeiddfhhidGbdeiiiiiffbddihiiiTdhhbiihhdbhhhdhhidbhibhieehdbddAfihiiiiiiieeCii
ideihhhiiififiTiiiiiiiiiTiiiihfhfiffbhhhAAfffffffddbdbbbbeddddddiieiiiiiiheebe
iifiihebhheebbAbhhhhiieeeddbhheei
```

```
seq_GFP   =
000000TG..G.....................................................................
....................................................A...........................
................................................................................
................................................................................
................................................................................
................................................................................
...............................................................A................
................................................................................
........................................0000000000000000000000000000000000000000
00000000000000000000000000000000000000000000000000000000000000000000000000000000
00000000000000000000000000000000000000000000000000000000000000000000000000000000
0000000000000000000000000000000000
seq_RFP   =
000000T...G.....C....A.........C................................................
................................................................................
................................................................................
................................................................................
................................................................................
.....................................................................AC.CCCA..CC
TC.GGCGGAA....TGC..GCG...TCC.GT..CAAGCG..A.AGCC.C..GA..CC..TCCTGT..G..TCTGA..
GCTCCCCCC.T.....CT..A.CCAGCCT.....ATT....CTTACCT.CTCACTAATCCCTTTCTACATA....T.
.CTC.CCC....CCT.G.......AC.A...GTA.G.0000000000000000000000000000000000000000
00000000000000000000000000000000000000000000000000000000000000000000000000000000
00000000000000000000000000000000000000000000000000000000000000000000000000000000
0000000000000000000000000000000000
```

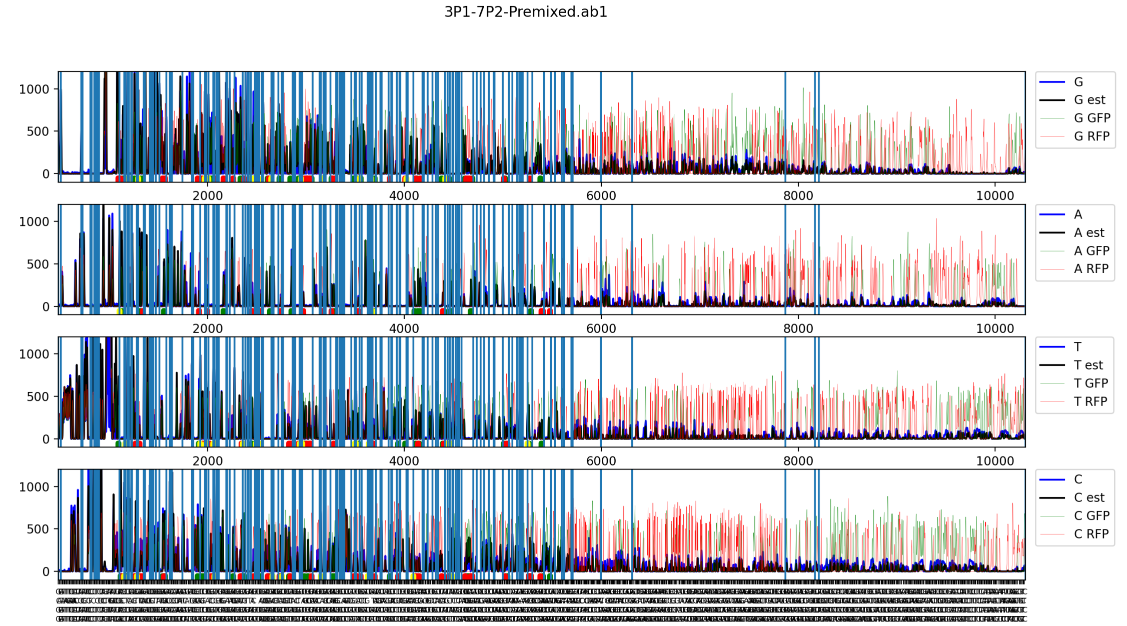


```
seq       =
GGAfTCAdebGdfbfCCef|ff|ffdifhdhhhi||h|ibi||ei||i||i|ii|ihed||fddb.....|...||.
|i.||...||....f|...iee|.|d.|.....b..|i|.hd.fbb.|ihTb||.d|.....|...||.|...||.|
d|....G|b.|...d......|.f.||.|..|||||.e|......||h...|..|e.......f||...|||.....
...f.e...||.|ee...|....ee.|||bfeef..|..||..|.i....|||||..f...iiff.|.|.ib...|.
i|....|i....|......f||.id..d|..i.|.....|.|.|.|i.|.ei.|........b..b.ie.i|...f
...fb..b...|....i..i...f.||f|...|...i.h......b.....d..if....i.ibGieheebieiiG
ihGTi..ibfhb.iiffefA|Abefieefei.ifhiieGhiGd.b.hdTibGeifTbbe.bbiidG..b.bdiiGi|
.dGidifTbiG.f.beedTbdhbeiddA.bibhfei.hbeeGf.bbdhfGdeiebdehebAGbGibbbe.beebihi
```

```
ddGef..iidiibbdddd|ibbfid..ef.ihedediA|e..ee..ihdedee.fiee..iie.iiii..hidebib
eei.dfhhidbf|eiidiifbed.heiid.hhh.e.ibbehhdhhid.bhbehbe.i.bbddAfh.eiiiide.iii
d.eifhh..h.hffihiiiiiii..iTiiiiifff.fffAhhhh.fff.f.dbf.bbbeiddi.ieiiiiihebbfi
iifihbhhheebAbehhifiieeddbhhheii
seq_GFP   =
00000000000000000T...........................................................
.............................................................................
.............................................................................
.............................................................................
.............................................................................
.............................................................................
.............................................................................
.............................................................................
...............................................................C.............
..G..G..C..GT....G..T....C..................................G................
..........A.CT..........C......T.....A....AC.....................000000000000
00000000000000000000000000000000
seq_RFP   =
00000000000000000T...A.......................................................
.............................................................................
.............................................................................
.............................................................................
.............................................................................
...................................................................CA..CCTA.C
TCCTC..AC.G..AA...AC...TG...TCC.GCT.CGCGAT....GGC..G..A.GCC..CCTAT..T.GGCCT..
..TGCCTG.C....CT...CCCGCCTG...ACCTA..CTTTCC.CTGTCATTCTCTTTCAATAAATT.......CCC
.TA..T..CAC........GAAC.T..A..GAAA.G...GA.T....ATTA.A......A............G..AA
AC..T.CTTC...GA.AACC..G.ACGT..TA..T..CCG.TTAC...CTTATAC...TCGTT...GAGAACA...A
..A.G......TAG.A...ATGG..TAAAGAAG.AG...CCGAC.........A..C..A.....000000000000
00000000000000000000000000000000
```

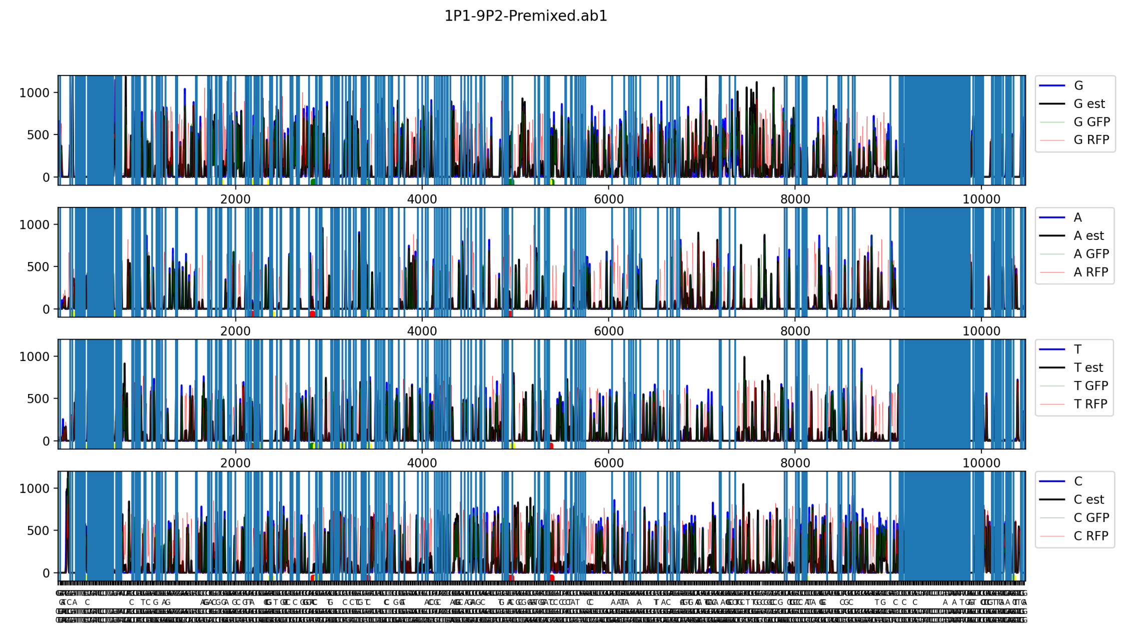


```
seq       =
dddGGG|G|dAff|||.||||f||||||||||h||||||||||||||||||||||||||||A|||CTTGT|CA.||C
||C||TG.||A.AG||AT.||||C||.|G.CAC|AA|||CAGTA|GA|CATG||A||GCGC.|..C||.|G.G||.|
```

```
||C.CA.||C|GA.|G.GTGC|.|.||.|.G||||C||GC....|||CG.|GC||.C....A||G.G|||TC.G.
.G.|.|...||G|||AGC|GC..||T||||||||C.|TG||.T|G|..A.|||||..|TCA|||G|T|C||..C|T
||GC.T||C...|ATGATAT||AC|TTG|GG|.|T..TA|.|.|A||C|A||T|G.GC...A.|AT|T.|.C|..C|
C.T||.A|TCG|TGCC|TT|A..|C||||...|TCA|.A.GG.GTC|..C..|.A||.C..|.|||..CG.GTCT.|
T.||.G.||.C||.|T|G|A|AA.A.GGTGCGCTCCTGGA||TA|.CTTCbG|i.|G.C|G|C|T|AAb|A||||TG
CT|C|Ti.|GTG|fC||.G|A|CG||T|AhbibCTGibCGCC|fhbGTC|Gde..bTCh|b|G||TbG|C..e.GC.
|d.G.Ae|TTG.CGGTdGd|Cb|iGA.CTdC..GeTCAG.TT||C|T.ded|.|fi|e||||biCi||bCCGGACb
.e|T|AA|TT|TG|||GiTT|e||C.C|G|CC|GCTCG|CC|GGATGGGi|CC|eCC|GG||AAChG||||||e|||
||||||h|||||||||||||||||||||||||||f|||||||h||||||f||||d|bb||i||||||eiiii|he||
|i||f||bb||||b|||||hif|i|||dbAhheii
seq_GFP   =
000000TG..G................................................................
...........................................................................
...........................................................................
...........................................................................
...........................................................................
...........................................................................
...........................................................................
...........................................................................
...........................................................................
...........................................................................
...........................................................................
............................000000
seq_RFP   =
000000TG..G................................................................
...........................................................................
...........................................................................
...........................................................................
...........................................................................
...........................................................................
...........................................................................
...........................................................................
...........................................................................
...........................................................................
...........................................................................
............................000000
```
